# Estimates of statistical significance for comparison of individual positions in multiple sequence alignments

**DOI:** 10.1186/1471-2105-5-106

**Published:** 2004-08-05

**Authors:** Ruslan I Sadreyev, Nick V Grishin

**Affiliations:** 1 Howard Hughes Medical Institute, and Department of Biochemistry, University of Texas Southwestern Medical Center, 5323, Harry Hines Blvd, Dallas, TX 75390-9050, USA

## Abstract

**Background:**

Profile-based analysis of multiple sequence alignments (MSA) allows for accurate comparison of protein families. Here, we address the problems of detecting statistically confident dissimilarities between (1) MSA position and a set of predicted residue frequencies, and (2) between two MSA positions. These problems are important for (i) evaluation and optimization of methods predicting residue occurrence at protein positions; (ii) detection of potentially misaligned regions in automatically produced alignments and their further refinement; and (iii) detection of sites that determine functional or structural specificity in two related families.

**Results:**

For problems (1) and (2), we propose analytical estimates of P-value and apply them to the detection of significant positional dissimilarities in various experimental situations. (a) We compare structure-based predictions of residue propensities at a protein position to the actual residue frequencies in the MSA of homologs. (b) We evaluate our method by the ability to detect erroneous position matches produced by an automatic sequence aligner. (c) We compare MSA positions that correspond to residues aligned by automatic structure aligners. (d) We compare MSA positions that are aligned by high-quality manual superposition of structures. Detected dissimilarities reveal shortcomings of the automatic methods for residue frequency prediction and alignment construction. For the high-quality structural alignments, the dissimilarities suggest sites of potential functional or structural importance.

**Conclusion:**

The proposed computational method is of significant potential value for the analysis of protein families.

## Background

Profile-based methods of sequence analysis use multiple sequence alignments (MSA) to extract information about conserved features of a protein family, which are impossible to decipher from a single sequence. Such methods increase both the sensitivity of homology detection and the quality of produced alignments [1-10], mainly due to more accurate scoring of similarity between sequence positions. Here, we address the problem connected to but different from the problem of scoring positional matches. We focus on detecting confident dissimilarities between profile positions that are suggested to be equivalent. In particular, we sought conservative P-value estimates for the comparison of individual columns in MSA. Such estimates have at least three practical applications: (i) evaluation and optimization of methods predicting propensities for residue occurrence at protein positions; (ii) detection of potentially misaligned regions in automatically produced alignments and their further refinement; and (iii) detection of sites of functional or structural specificity in two related families.

Statistical analysis at the level of individual MSA positions may be used to compare residue frequencies predicted from some model to the actually observed residue usage at the given position in sequence homologs. The model may represent, for example, a method for *in silico *sequence design that generates native-like sequences from a structural template. Detection of discrepancies between the model and the real data would assist the analysis of the model's performance and its further improvement. To our knowledge, such statistical assessment has not been proposed up to date.

Several approaches have been proposed to detect potential regions of low alignment quality in sequence-sequence and sequence-profile alignments. These approaches range from identifying low-scoring regions in pairwise alignment [11] to more complicated schemes: comparing scores of the given alignment and the optimal alignment where this position is omitted [12], or analyzing the consistency of a given position among different alignments produced with various parameters of alignment construction [13,14]. For multiple sequence alignments, positional residue conservation was proposed as a measure to detect potentially misaligned regions of high variability [15,16]. Cline and co-authors [17] compared several methods for positional evaluation of sequence-profile alignments and recommended the approach based on the analysis of near-optimal alignments [13,14]. However, detection of potentially misaligned regions in profile-profile alignments has not been addressed before.

When the analyzed alignment is highly reliable, detecting positions of significant dissimilarity may reveal sites that determine functional or structural specificity of otherwise similar proteins. Several approaches have been proposed that use comparison of multiple sequence alignments in order to predict such sites [18-21]. However, these methods do not involve explicit estimation of statistical significance. Bejerano [22] has recently proposed a promising algorithmic approach to the exact P-value computation, which allows for a faster enumeration of possible outcomes. Despite a significant improvement in the computational efficiency, the algorithm still requires a considerable time to process realistic data in 20-dimensional space of residue frequencies.

In this work, we consider approximate analytical estimates of P-value in two settings: (1) comparison of an alignment column to an emission vector of residue probabilities, and (2) comparison of two alignment columns. These estimates allow detecting cases where the null hypothesis (assumption of similarity) can be confidently rejected. We performed simulation experiments that show consistency of the estimates with the statistical model, and applied our method, PEAC (P-value Estimation for Alignment Columns), to the analysis of real MSA.

## Results

### Theory

As the statistical null model of a multiple alignment column, we assumed independent random draw of residues according to a vector of emission probabilities. We represented randomly generated columns by vectors of residue counts ***n***, with total count *N *equal to that of the real alignment column under evaluation.

#### Statistical significance of similarity between a multiple alignment column and a vector of emission frequencies

##### Null hypothesis **H**_**0**_^**(1)**^

given alignment column (vector of residue counts n*) is generated by given vector of emission probabilities ***f***. If this hypothesis is rejected, then the set of emission probabilities is inadequate for the description of the residue content in this alignment column.

The assumed null model of random columns corresponds to a multinomial form of *ρ*(***n ***| ***f***), which is difficult for analytical consideration. To calculate the P-value, we use the multivariate Gaussian approximation of the multinomial distribution, based on the assumption of large statistical samples (large total residue counts *N *in the generated columns):


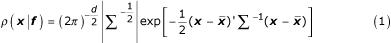


where ***x ***= {*x*_*i*_} is a random *d*-dimensional vector of residue counts of size 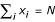, ***f ***is emission vector of residue frequencies, 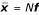 is the mean vector of residue counts, *Σ *= ||cov(*x*_*i*_, *x*_*j*_)|| is the covariance matrix. This approximation of p.d.f. allows for the analytical expression for the P-value (Appendix 1 [see Additional file 1 ]):


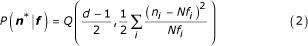


where 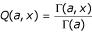 is a regularized gamma-function, *d *is dimensionality of vector ***f***. Thus, P-value is described by a *χ*^2 ^distribution with (*d *- 1) degrees of freedom.

###### Random simulation shows consistency of P-value estimates with null model

**Figure 1:**
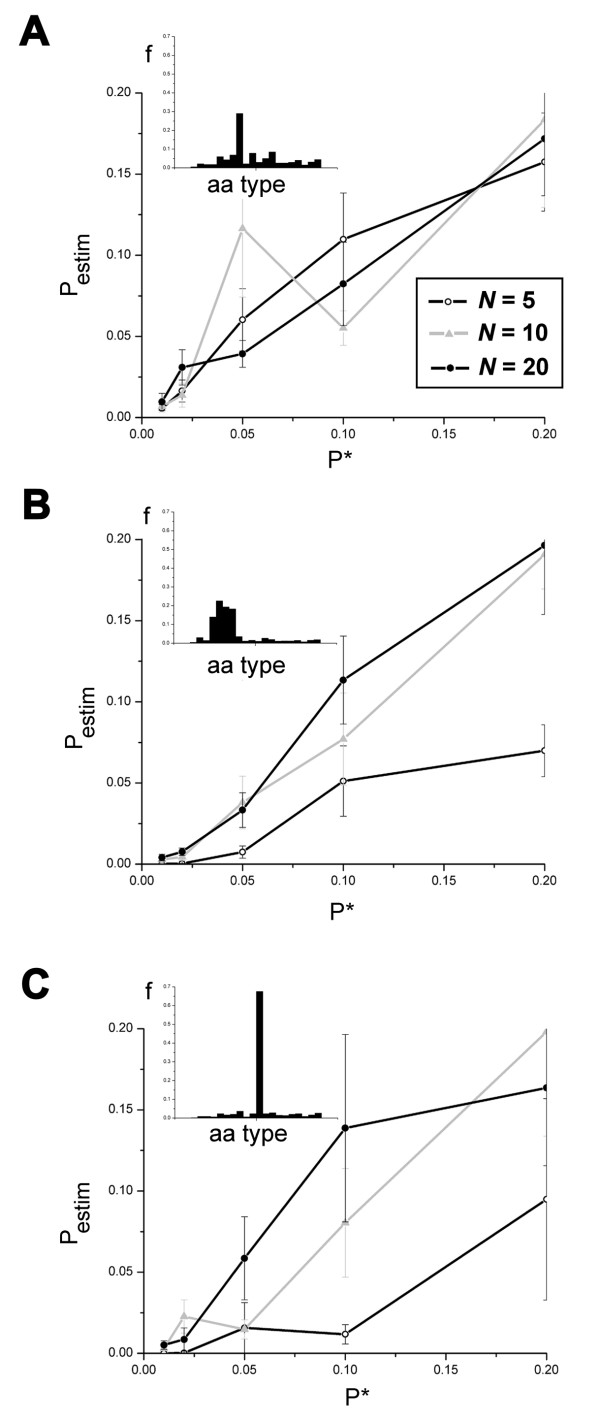
**P-value estimates for comparison between alignment columns and residue frequency vectors: random simulations show general consistency with null model. **(A-C) For different emission vectors based on real alignment columns, large sets of random columns of three different sizes *N *were generated, and P-value estimates by PEAC (P_estim_) were plotted against experimentally estimated P-values (P*, see text for details). Sets of emission frequencies are shown as bar graphs in inserts.

In order to analyze whether the Gaussian approximation allows reasonable P-value estimates, we performed extensive random simulations and tested consistency of P-values based on this approximation (formula (2)) with P-values based on the multinomial model. In particular, we used a set of residue frequencies ***f ***= {*f*_*i*_}_1_^20 ^to generate a large number *Ω *= 10^7 ^of random columns of a fixed size *N*, i.e. *Ω *sets of *N *residues drawn randomly according to probabilities *f*_*i*_. For each random column, residue counts ***n ***= {*n*_*i*_}_1_^20 ^were derived and the multinomial probability of its generation was calculated as 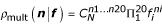, where 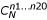 is the multinomial coefficient. All *Ω *generated columns were sorted by *ρ*_mult _in the ascending order. For a given P-value P*, the column number *Ω*P_0 _was chosen from this sorted list. This column corresponded approximately to the multinomial P-value P*. This P-value was compared to our estimate *P*_estim _(formula (2)) calculated for the chosen column in the Gaussian approximation of multinomial distribution. For each value P* we performed 10 independent simulations and plotted average values of *P*_estim _against P*, which showed their general consistency. Figure 1 illustrates the results for three typical sets of emission frequencies ***f ***derived from real alignment columns, and for three typical column sizes *N*. The accuracy of estimates becomes poorer for lower column sizes and more skewed frequency sets (Fig. 1A). However, even in such cases the accuracy of *P*_Gauss _within orders of magnitude is sufficient for the purpose of detecting the pronounced dissimilarities with *P *<< 0.05. Thus, the error introduced by Gaussian approximation still allows the use of P-value estimates under the initially assumed null model of random columns.

#### Statistical significance of similarity between two columns of multiple alignments

##### Null hypothesis ***H***_**0**_^**(2)**^

two observed columns m* and n* are generated by a single vector of emission probabilities. As the prior distribution of emission vectors, we use the maximum likelihood (ML) estimate based on m* and n*. Such prior should produce the conservative upper estimate of the P-value. Rejection of hypothesis *H*_0_^(2) ^would mean that the two alignment columns are highly dissimilar.

The P-value for this hypothesis is calculated in three steps:

a). Given the two vectors of residue counts {***n***, ***m***}, we produce the ML estimate of the p.d.f. for emission vectors ***f ***that can generate both columns simultaneously. We assumed a simple form of multivariate Gaussian distribution and calculated ML estimates of its mean 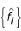 and variance values 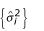 (formulae B5).

b). We use this p.d.f. *θ*(***f***) as the prior to calculate the posterior probability *ρ*(***n***, ***m *| ***θ*(***f***)) that a pair of random columns {***n***, ***m***} is produced by any single emission vector ***f***. Similarly to problem 1, we use multivariate Gaussian approximation of the multinomial distribution that assumes large total residue counts in the generated columns. The posterior probability density can be calculated as

*ρ*(***m,n*** | *θ*(***f***)) = *∫ρ* (***m,n*** | ***f***) *θ* (***f***) *d****f***     (3)

c). Using (3), we calculate P-value as the integral (Appendix 2 [see Additional file 2]):


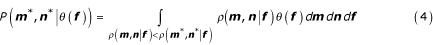


This value can serve as the upper estimate of the P-value, since the prior distribution *θ*(***f***) is a ML estimate based on the observed alignment columns. The partial integral *∫ρ* (***m,n*** | ***f***) *d****m ****d****n*** can be calculated analytically for any emission vector ***f***, but analytical calculation of full integral (4) is problematic. However, an approximate estimate of this value would suffice, since (i) expression (4) already contains approximations introduced by estimates of *θ*(***f***), *ρ*(***n***, ***m *| ***θ*(***f***)) and *ρ*(n*, m* | *θ*(***f***)); and (ii) we are interested in a conservative estimate of the upper P-value limit. Hence, we calculate an approximate upper estimate of P-value (Appendix 2 [see Additional file 2]):


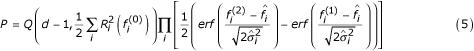


where *erf*(*x*) is error function, and


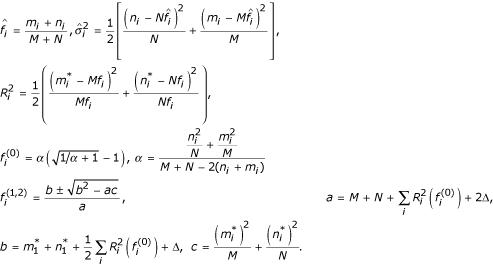


###### Random simulation shows consistency of upper P-value estimates with null model

**Figure 2:**
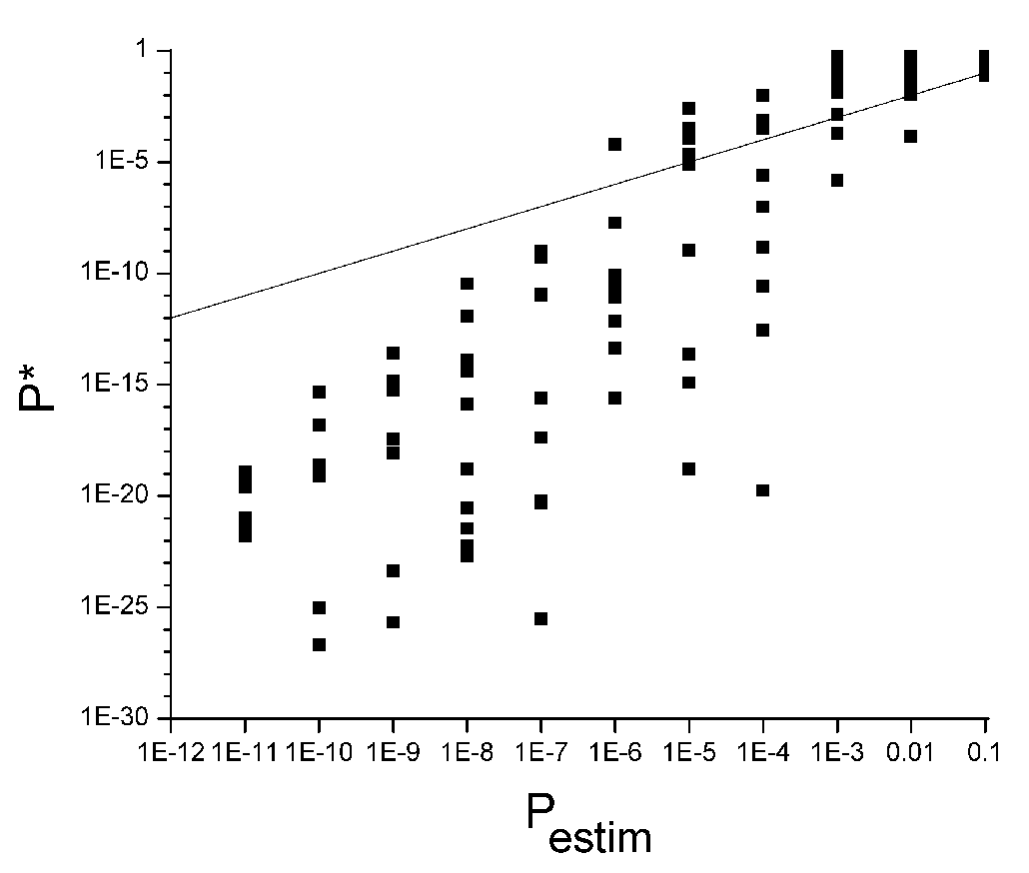
**Upper estimates of P-value for comparison between two alignment columns: random simulation tests for consistency with null model. **Using a random emission vector, a pair of columns was randomly generated that corresponded to a specified value of the PEAC estimate of (*P*_estim_). P-value for the generation of such a pair by the used emission vector (P*) was plotted against *P*_estim_. See text for details.

To assess the consistency of our estimates with the null model, we performed the following simulation experiments. A random emission vector of residue frequencies ***f ***was used to produce a column of size *N *by random draw according to these frequencies. Having the vector residue counts ***n ***in this column, we produced another vector of counts ***m ***that made our estimated P-value *P*_estim_(***m***,***n***) equal to the specified value *P*_0_. To produce this vector, we considered sets of residue counts as points in multidimensional and randomly chose a straight line passing through the point ***n***. On this randomly directed line, we found the point ***m ***as the solution of equation *P*_estim_(***m***,***n***) = *P*_0_, where *P*_estim_(***m***,***n***) is defined by formula (5). Thus, we generated a pair of columns that corresponded to the specified P-value according to the PEAC estimate. We compared this estimate to the actual P-value P* calculated for the generation of ***m ***and ***n ***by the original vector ***f***. As shown by the plot of P* against *P*_estim _(Fig. 2), a particular estimate of P-value may correspond to various actual values P*. However, for low P-values, i.e. for the range of our interest, PEAC systematically produces *P*_estim _higher than actual values P*, as expected from the upper P-value estimates. These conservative estimates ensure the absence of false positive results among detected cases of significant dissimilarity.

We developed P-value estimates for the following null hypotheses (see Theory): (1) a given alignment column is generated by a given set of emission residue frequencies; and (2) two given alignment columns are generated by a single set of residue frequencies. We applied both types of estimates to the analysis of real multiple alignments, detecting cases of significant dissimilarity where the null hypotheses were confidently rejected.

### Application

#### Comparison of an alignment column to a frequency vector

**Figure 3:**
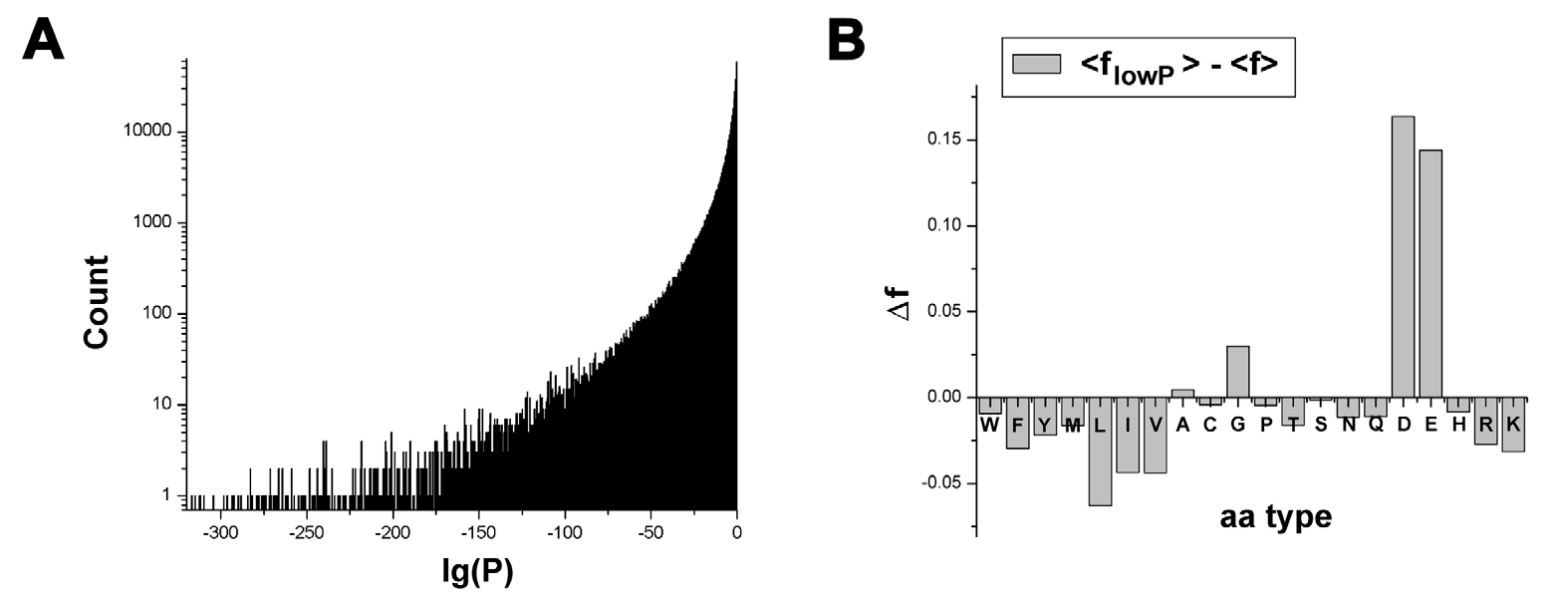
**Comparison of residue frequencies in multiple alignments of sequence homologs to the structure-based frequency predictions. **A. Histogram of P-values (log-log scale). B. Difference in residue frequencies between 0.3% alignment positions with lowest P-values and the whole population. Frequencies of glutamate and aspartate show the greatest elevation.

Using our method, we assessed the consistency between predictions of residue frequencies based on structural considerations, and the frequencies in multiple alignments of sequence homologs. Specifically, we prepared a dataset of 1695 PDB structures and made predictions of residue propensities at each position, based on local structural environment. In parallel, the sequences corresponding to these structures were used as queries for PSI-BLAST searches, and profiles of detected confident sequence homologs were constructed (see Methods). The effective residue frequencies at profile positions were compared to the structure-based predictions, and P-values for each position were estimated using PEAC. The histogram of produced P-values for all positions is shown in Fig. 3A. These P-values ranged widely between 10^-320 ^and 1.0, with the median being approximately 0.01.

To analyze the cases of most pronounced discrepancy between our structure-based predictions and residue frequencies observed among sequence homologs, we chose ~1000 protein positions (0.3% of the whole dataset) that had lowest P-values (*P *< 10^-100^). These sites were located mainly in the secondary structure elements, most frequently at their ends, and corresponded to unusual local distortions of 3D conformations. We compared residue content in the corresponding subset of alignment columns to the whole dataset. As shown in Fig. 3B, alignment positions with low P-values demonstrated unusually high average frequencies of negatively charged residues, glutamate and aspartate.

**Figure 4:**
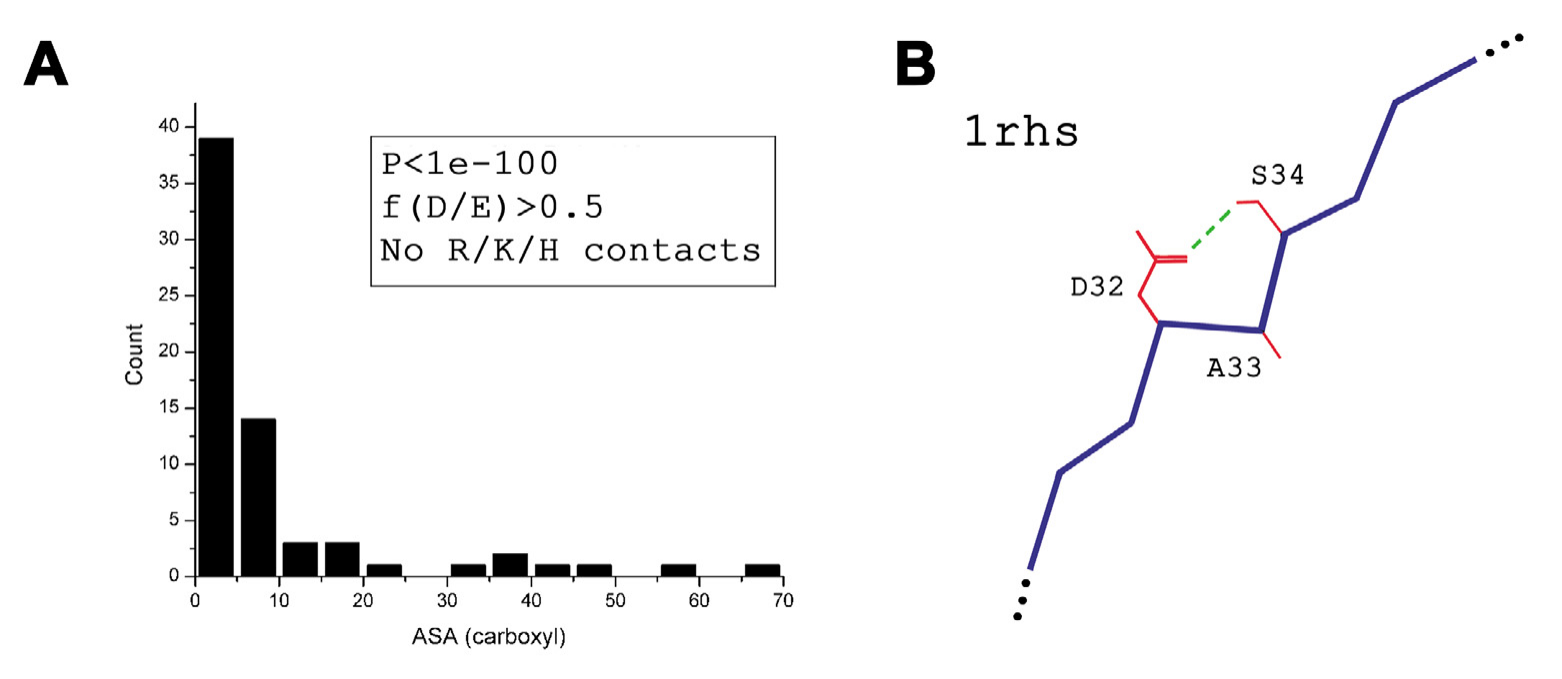
**Structural environments for protein positions that show discrepancy between observed residue frequencies and their structure-based predictions. **A. Histogram of accessible surface areas (ASA) for D/E residues at the positions that show largest discrepancy (*P *< 10^-100^), correspond to elevated frequencies of D/E among sequence homologs (effective frequencies more than 0.5), and do not contact with positively charged R, K or H residues. B. An example local structural distortion caused by contacts of buried acidic residues. A fragment of structure (PDB ID 1 rhs) with aspartate beta bulge is shown. Backbone is colored with blue, sidechains with red. Contact formed by aspartate D32 through the side chain oxygen is marked.

For a more detailed analysis, we considered the subset of 145 alignment positions with *P *< 10^-100 ^that contained highly conserved D or E, and inspected corresponding D or E residues in tertiary structures. The vast majority of these residues were buried, as was indicated by the accessible surface area (ASA) of their carboxyl caps (data not shown). When we excluded glutamates and aspartates whose charge could be neutralized by contacts with positively charged arginine, lysine or histidine, the remainig portion of the set was still comprised of mostly buried residues (Fig. 4A). These buried residues with acidic side chains did not form salt bridges with basic side chains, which is the most typical way of neutralizing a charge in hydrophobic environment. Inspecting these positions manually, we found a less usual mode of charge neutralization, which involves contacts with other polar residues. A typical example of such conformation is a motif classified in the I-site database [23,24] as aspartate beta bulge, located in the middle of a beta strand in bovine rhodanese (thiosulfate:cyanide sulfurtransferase, PDB ID 1 rhs, Fig. 4B). The contact between side chain oxygen of D32 and S34 distorts the regular beta-strand conformation. Our scheme of structure-based frequency prediction considered only most common classes of local conformations that involve nearest neighbor residues. This scheme could not account for less usual residue contacts and therefore failed to predict a high conservation of buried acidic residues at this position, which may have functional or structural importance [25] In summary, this application of our method assists detecting positions with discrepancies between the predicted and naturally occurring residue frequencies. A detailed analysis of these positions may highlight shortcomings of a predicting scheme and suggest possible directions for improvement.

#### Comparison of two alignment columns

Statistical comparison of two MSA positions may be used in two applications. (i) In automatically produced alignments of sequences or structures, consideration of profiles of confident homologs helps to detect inconsistencies. According to our observations, these inconsistencies are caused mainly by alignment errors. (ii) In the high-quality structure based alignments, where structural equivalence of residues is confident, the low P-values may indicate functional specificity of spatially aligned residues.

##### Detection of errors in sequence alignments

As an example of application (i), we evaluated our method by ability to predict erroneous residue matches produced by an automatic sequence aligner (ClustalW [26]), as compared to the high-quality reference alignments in a manually curated database, BaliBase [27]. For each BaliBase alignment, we (1) extracted individual sequences and generated their ClustalW alignment; (2) for the top and the bottom sequences of BaliBase alignment, produced MSAs of their homologs detected by PSI-BLAST; and (3) used the resulting alignment pair to estimate P-values for the sequence positions matched by ClustalW.

**Figure 5:**
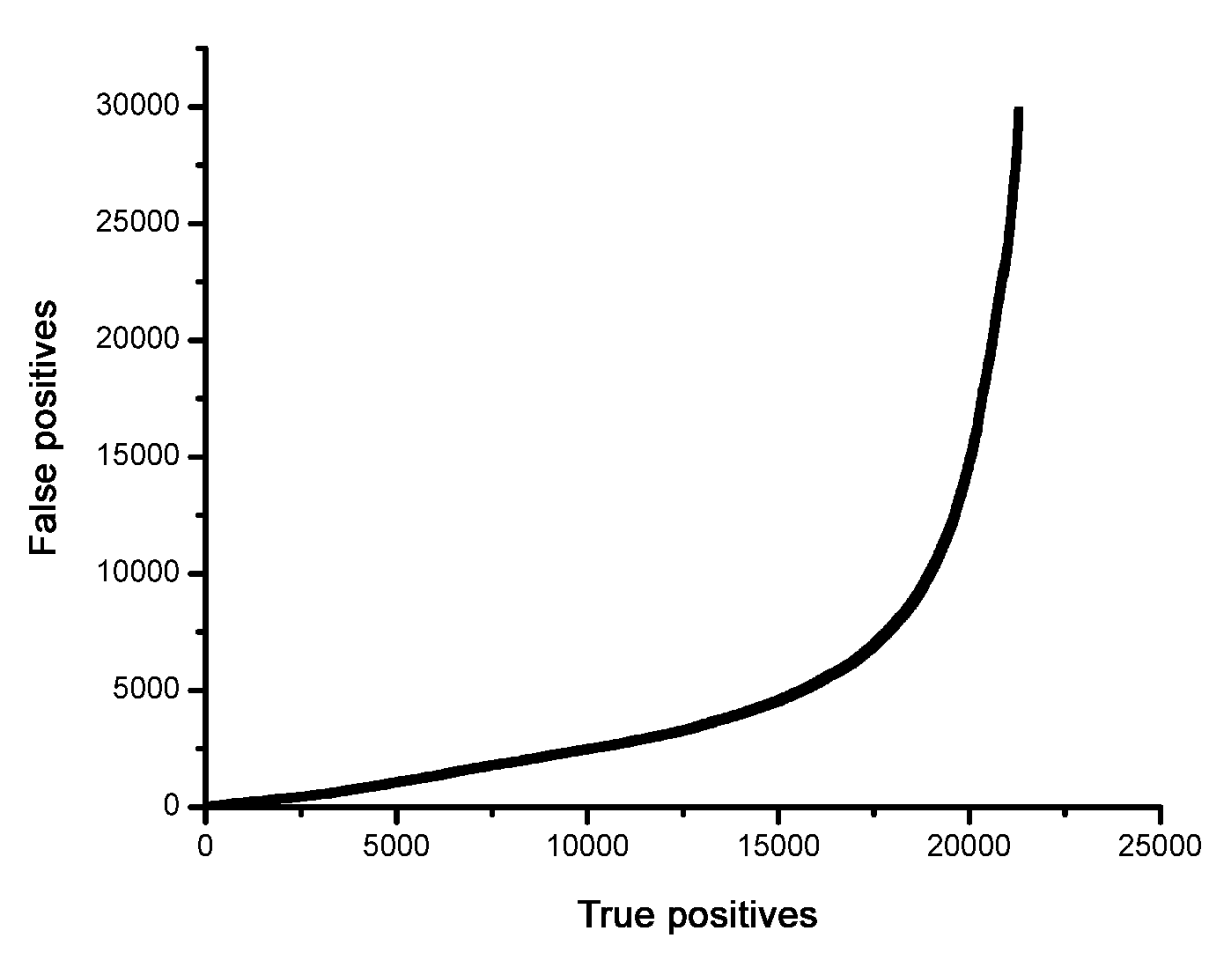
**Sensitivity plot for the detection of errors in automatically produced multiple sequence alignments. **The set of positional matches in ClustalW alignments was ranked by ascending P-values for corresponding profile positions and classified as true or false predictions. True positive predictions are errors in ClustalW alignments compared to the BaliBase reference. False positive predictions are correst position matches consistent with BaliBase.

We then sorted all ClustalW positional matches by ascending P-values and classified them as true or false predictions of ClustalW errors. For our purpose, the ClustalW matches different from those in BaliBase were considered true positive predictions; whereas correct matches were considered false positives. Having the ranked list of true and false positive predictions, we generated sensitivity curve (plot of the number of true positives vs. the number of false positives, Fig. 5). The curve shows the degree of discrimination between erroneous and correct positional matches. Among the top 1000 predictions, the method generated 151 false positives. Up to ~17,000 true positives, the rate of false positive predictions is slowly growing, then this rate considerably increases. This point approximately corresponds to the P-values of ~10^-2^.

##### Detection of evolutionarily unrelated positions in structure-based alignments

We applied our method to detect profile dissimilarity between protein positions that are aligned by an automatic structure based method. Specifically, we (1) collected pairs of protein domains that are structurally similar according to the DALI alignments [28] in the FSSP database [29,30], (2) for each of these proteins, produced MSA of homologs detected by PSI-BLAST, and (3) used the resulting pairs of alignments to estimate P-values for the positions matched in FSSP. We used two sets of the FSSP domain pairs, with different sequence identities between the domains: 25 ± 1% (the upper limit of "twilight zone", which generally allows for homology detection and alignment construction based on sequences alone [31,32])., and 15 ± 1% (a lower range of identity, where structural alignment is more difficult to reproduce by sequence comparison).

**Figure 6:**
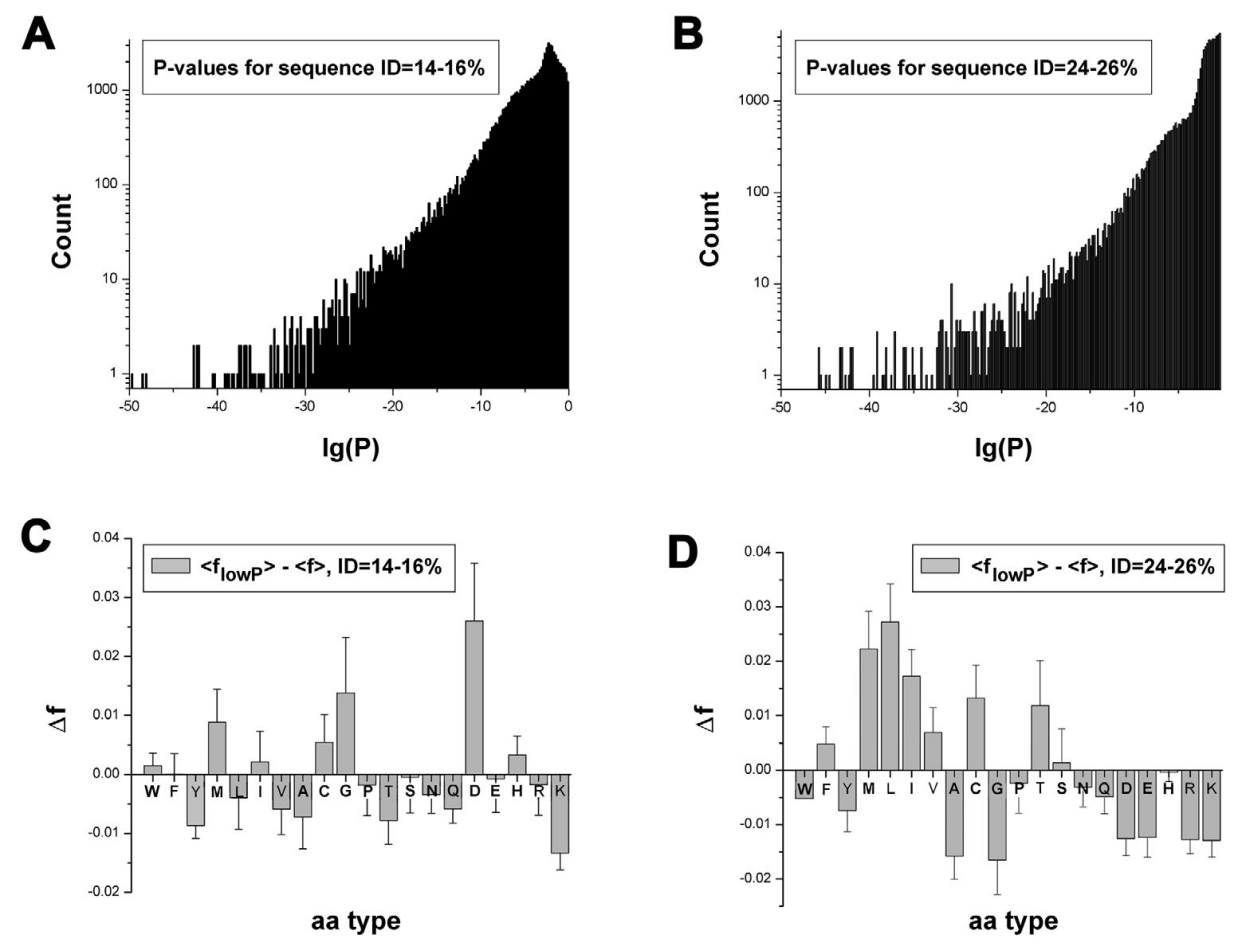
**Detection of structurally aligned protein positions that correspond to dissimilar profile positions. **(A, B) Histograms of P-values for FSSP alignments between domains of different sequence identity (log-log scale): A. Sequence identity 15 ± 1%. B. Sequence identity 25 ± 1%. (C, D) Difference of residue frequencies at alignment positions with lowest P-values and the whole population: C. Sequence identity 15 ± 1%, *P *< 10^-14 ^. D. Sequence identity 25 ± 1%, *P *< 10^-18^.

Figures 5A,5B show the histograms of P-values produced for pairs of profile positions that correspond to structurally aligned residues. The distributions of P-values were different for the two ranges of sequence identities. For identities around 15% (Fig. 6A), the histogram had maximum at approximately 0.5 10^-2 ^and the median was approximately 0.1 10^-2^. For identities around 25% (Fig. 6B), the maximum was above 0.1 and the median was approximately 2.5 10^-2^, which shows much better consistency between structure-based and profile-based alignments.

To analyze the most dissimilar profile positions, in each dataset we chose 0.3% of position pairs that had lowest P-values (639 pairs for identities 15 ± 1%, and 760 pairs for identities 25 ± 1%). Using Insight II suit for molecular modeling and simulation (Accelrys), we performed a detailed manual analysis of structural superposition for a portion of the corresponding structural alignments. We found that the majority of inspected positions were apparently misaligned. Approximately 80% of these residues were located within 5 positions from a gap introduced in the structural alignment. Vicinities of gaps generally correspond to less similar fragments of aligned structures, which are more difficult to superimpose and where alignment errors can occur more frequently.

We considered residue contents of the MSA columns corresponding to these low-P-value position pairs, and compared these contents to the average residue frequencies in the whole MSA datasets. In the set corresponding to 15 ± 1% sequence identity, the most pronounced difference was a higher frequency of aspartate at the position pairs with low P-values (Fig. 6C). In the set for 25 ± 1% identity, the low-P-value positions had higher frequencies of methionine, leucine and isoleucine (Fig. 6D).

We further concentrated on the aligned structural positions that showed unusual residue frequencies in the corresponding MSA columns. In the set corresponding to 15 ± 1% sequence identity, we considered positions with highly conserved aspartate, whereas in the set corresponding to 25 ± 1% sequence identity, we considered positions with high combined frequency of methionine, leucine and isoleucine. In an attempt to exclude apparently misaligned positions, we considered only those positions that were distanced more than 5 residues from gaps in the FSSP structural alignment. We selected and manually analyzed 16 of such positional matches. However, even among these selected matches most of the discrepancies were still caused by apparent alignment errors: 10 cases corresponded to structural misalignments (usually due to a shift in 1 position), and 3 cases were caused by biased residue frequencies at profile positions, due to errors in PSI-BLAST alignments of sequence homologs. The remaining 3 position pairs did not involve apparent errors of either DALI or PSI-BLAST. These pairs might represent real differences in residue preferences at structurally equivalent positions.

**Figure 7:**
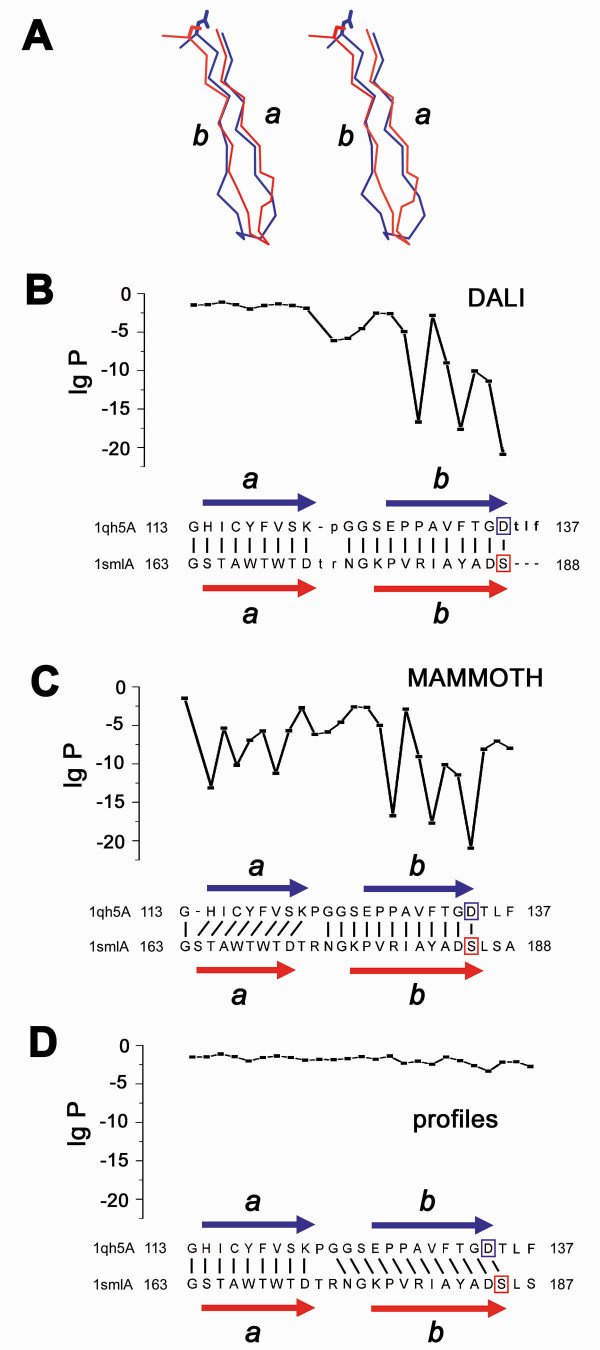
**Two sources of discrepancies between structure-based and profile-based position similarities: misalignment and structural or functional specificity. **A. Manual alignment of fragments of human glyoxalase II (PDB ID 1qh5A) and bacterial penicillinase (PDB ID 1sml). Strands ***b ***are shifted by one register, so that structurally similar residues correspond to different sequence positions. In these strands, sidechains are shown for the residues involved in Zn binding (D134 and S 185, respectively). (B-D) Alignments produced for these fragments by different methods, with corresponding positional P-values. Secondary structure elements (beta stands ***a ***and ***b***) are shown with arrows. Structurally equivalent residues are connected with lines, residues involved in Zn binding are boxed. B. DALI correctly reproduces structural alignment of strands ***a***, resulting in relatively high positional P-values in this region. Alignment of strands ***b ***is also structurally correct, but their shift in the two structures results in discrepancy between structural alignment and profile content, as reflected by low P-values. C. MAMMOTH misaligns strands ***a ***by one register. Low P-values indicate significant profile dissimilarity between aligned positions in both regions ***a ***and ***b***. D. Profile-based alignment is in good accord with profile content, as shown by higher P-values for regions ***a ***and ***b***. Note that alignment in region ***b ***differs from the structure-based alignment shown in B.

Figure 7 shows two examples of low P-values for protein regions that were superimposed by automatic structure aligners. The first example illustrates a typical case of apparent misalignment. The second example represents a case that is observed much rarer among automatic structural alignments: the structure superposition is correct but inconsistent with sequence-based similarity. Such inconsistency might represent a change in the structural role of evolutionary related positions. Human glyoxalase II (PDB ID 1qh5) [33] and bacterial metallo-beta-lactamase L1 (penicillinase, PDB ID 1sml) [34] belong to different families of the SCOP metallo-hydrolase/oxidoreductase superfamily. Although these proteins share only 16% sequence identity, their structures are highly similar (DALI Z-score 16.3). Both glyoxalase II and penicillinase bind two zinc atoms at similar locations. Fig. 7A shows a manual structural alignment of their fragments, beta hairpins that contain residues involved in Zn binding (D134 in 1qh5A and S185 in 1sml). These residues have similar orientation of their sidechains (shown in Fig. 7A, strand ***b***). In glyoxalase II, D134 binds zinc atoms directly [33], whereas in penicillinase, S185 is linked with zinc through a water molecule [34]. Figures 7B,7C and 7D show sequence alignments of these regions and corresponding positional P-values based on automated structure comparisons by DALI (Fig. 7B) and MAMMOTH [35] (Fig. 7C), and on the comparison of sequence profiles (Fig. 7D).

Alignments of strands ***a ***illustrate superposition errors as the typical source of low P-values for automatic structural alignments. DALI (Fig. 7B) constructed the correct alignment, which was the same as the manual structure alignment (Fig. 7A) and profile-based alignment (Fig. 7D). This alignment corresponded to high positional P-values. MAMMOTH (Fig. 7C) apparently misaligned strands ***a ***by introducing a one-position register shift, which resulted in the low P-values for this region (Fig. 7C).

Structural alignments of strands ***b ***represent a rare example of an automatic alignment that corresponds to low positional P-values and yet is correct from the structural viewpoint. Both DALI and MAMMOTH produced the alignment consistent with the confident manual superposition. This structural superposition correctly aligns zinc-binding residues (D134 in 1qh5A and S185 in 1smlA). However, such alignment corresponds to low positional P-values, indicating a significant difference between structure-based and sequence-based position similarity. The optimal profile-based alignment (Fig. 7D) has a one-residue shift that dramatically increases positional P-values in this region, but is inconsistent with the topology of the beta strands and zinc-binding sites. Such a shift might represent a change in the structural roles of related protein positions in remote homologs. Indeed, zinc binding role in penicillinase 1smlA is transferred from residue D184, which is related to the zinc binding D134 of glyoxalase II (1qh5A [33]), to the neighboring S185 [34]. Thus, in the case of a high-quality structural alignment, low positional P-values may indicate evolutionary dissimilarity of spatially superimposed residues. Such cases, however, comprised a minor portion among automatic structure-based alignments and were overwhelmed by the cases of misalignment.

##### Prediction of structurally and functionally specific protein positions

**Figure 8:**
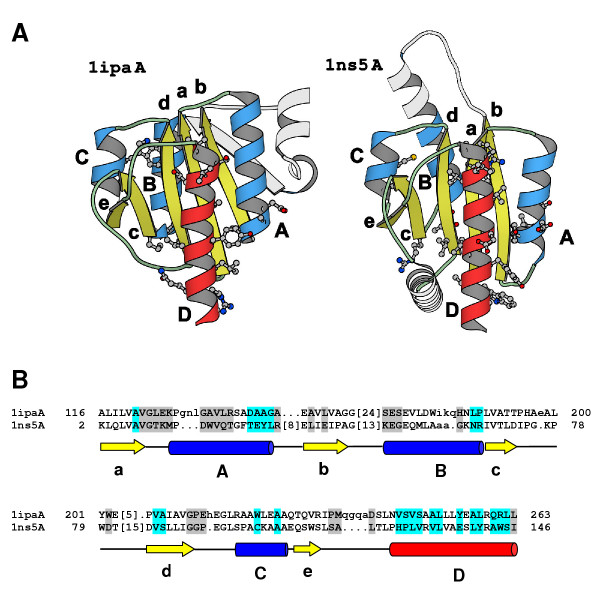
**Analysis of positional differences in protein families can reveal potential sites of functional specificity. **Comparison of structurally equivalent positions in the multiple alignments of sequence homologs for two distant relatives, 2'-O ribose methyltransferase from *T. thermophilus *(PDB ID 1ipaA) and hypothetical protein Ybea from *E. coli *(PDB ID 1ns5A). A. The ribbon diagrams of the structurally similar domain fragments (1ipaA, residues 116–263, and 1ns5A, residues 2–146) drawn by BobScript, a modification of the MolScript program [50]. Similar secondary structure elements are colored in blue (*α*-helices) and yellow (*β*-strands). The C-terminal helix D, which is involved in dimerization, is highlighted in red. Ball-and-stick models of sidechains are shown for the residues that (i) have C^*α *^distance less than 2A in the superimposed structures of the two domains, and (ii) correspond to significantly different positions in the multiple sequence alignments of the compared families (P < 0.01). In the ball-and-stick models, C, N, O, and S atoms are shown in gray, blue, red, and yellow, respectively. B. Structure-based sequence alignment of the two domains. The *α*-helices and *β*-strands are displayed as arrows and cylinders, respectively. Highlighted: the protein positions that have significantly different residue content in the two corresponding multiple alignments of sequence homologs detected by PSI-BLAST. Cyan, spatially close protein positions, with C^*α *^distance in the superimposed structures less than 2A; gray, position pairs with higher C^*α *^distances.

As an example of possible predictions of functionally specific regions, we considered positions in multiple alignments of sequence homologs for two structurally similar but evolutionary divergent proteins: RNA 2'-O ribose methyltransferase from *T. thermophilus *[36] (PDB ID 1ipaA) and hypothetical *E. coli *protein Ybea (PDB ID 1ns5A). These proteins possess the same *α*/*β *knot fold but belong to different SCOP families, SpoU-like RNA 2'-O ribose methyltransferase and Ybea-like, respectively. Using manually curated structure-based alignment of the two proteins and MSAs of their homologs detected by PSI-BLAST, we considered structurally equivalent positions that were well aligned in space (C^*α *^distance less than 2 A, Fig. 8) but showed significantly different residue contents in the MSAs (*P *< 0.01). We found 24 such positions, the majority being concentrated in the region of dimer interface, which includes the 'knotted' C-terminal helix D (Fig. 8A). In RNA 2'-O ribose methyltransferase, this region is suggested to be crucial for the molecular dimerization [36]. Positions detected in other regions mostly correspond to buried residues of hydrophobic core. The discrepancies in residue content at these positions may reflect different structural solutions for the sidechain packing within the core, as in the case of buried residues in helix C (W225 in 1ipaA *VS *C112 in 1ns5A, Fig. 8). Thus, the detected positional differences between SpoU-like and YbeA-like families highlight the functional importance of the 'knotted' C-terminal helix and may suggest a family-specific mode of dimerization and dimer activity for the hypothetical protein YbeA.

## Discussion

Here, we applied the concept of statistical significance to comparison of single positions of multiple sequence alignments. We proposed rigorous problems of the P-value estimation for the comparison of an alignment column to an emission frequency vector; and for the comparison of two alignment columns. We suggested approximate analytical solutions to these problems and applied the resulting P-value estimates to the analysis of protein families.

### Comparison of an alignment column to an emission frequency vector

Using our method, we compared residue conservation among sequence homologs and residue propensities predicted from local structural environment. The cases of the highest discrepancy between observed and predicted residue frequencies were enriched with positions containing conserved buried residues D/E. Many of these acidic residues do not form a salt bridge with basic side chains, but use contacts with polar residues to neutralize the negative charge in hydrophobic environment. Surveys of such contacts formed by aspartate residues were previously performed by Singh and Thornton [37] and by Fiser *et al. *[25]. The observed residue conservation may indicate the importance of such motifs for protein structure or function. The structure-based statistic for the prediction of residue propensities used only common classes of structural environments and considered closest neighboring residues in polypeptide chain. Hence this statistic was unable to predict the found conservation of buried glutamate and aspartate. Detection of such contradictions between predicted residue propensities and actual residue frequencies in MSA has three main implications. First, analysis of these contradictions can assist evaluation and further optimization of the predicting schemes, including knowledge-based potentials [38-41] or environment-specific substitution tables [42,43]. Second, the patterns of atypical relations between residue conservation and structural conformation may point to local motifs of potential structural or functional significance. Third, such atypical patterns, which are unlikely to coincide in two proteins by chance, may serve as signatures for homology detection.

### Comparison of two alignment columns

We used our estimates to assess similarity between MSA positions. First, we evaluated our method by detection of erroneous residue matches produced by an automatic sequence aligner, ClustalW [26]. The evaluated automatic alignments were compared to the high-quality reference alignments in a manually curated database, BaliBase [27]. Second, we estimated P-values for MSA positions corresponding to structurally aligned residues in the FSSP database of automatic structure based alignments [29,30]. We found that among detected cases of highest dissimilarity, the vast majority was caused by local structural misalignment. Correction of such alignment errors typically produced an increase of P-values (see results for strand ***a ***in Fig. 7C*VS *Fig. 7B). These results suggest a potential value of the method for the detection of misaligned regions in automatic alignments.

In our set of FSSP structural alignments, correctly aligned sites of low P-value were very rare. Such sites correspond to structurally equivalent positions that have different residue content in two related families. To illustrate the detection of such family-specific protein positions, we used a high-quality manually curated structural alignment of distantly related SpoU-like and YbeA-like families of the same *α*/*β *knot fold (Fig. 8). In addition to specific preferences for sidechain packing in the hydrophobic core, the statistically significant positional differences emphasized the importance of the 'knotted' helix (Fig. 8A), which is essential for dimer formation [36]. These differences may suggest a family-specific mode of dimerization and dimer activity for the hypothetical protein YbeA.

## Conclusions

We proposed P-value estimates to assess statistical significance for (1) comparison of a single position in a multiple alignment to a set of emission residue frequencies; and (2) comparison of two alignment positions. Computational implementation of these estimates showed its potential value for several important tasks in sequence analysis: (i) evaluation and optimization of methods predicting propensities for residue occurrence at protein positions, such as protocols for *in silico *sequence design; (ii) detection of potentially misaligned regions in automatically produced alignments and their further refinement; and (iii) detection of sites that determine functional or structural specificity in two related families.

## Methods

### Calculation of effective residue counts in multiple alignments

Effective residue counts at alignment positions were calculated based on the PSIC [44] method. We calculated 21 counts *n*_*eff*_^*PSIC *^for each symbol in the alignment column (including gaps, which are considered the 21^st ^symbol), and then applied the following transformation [16]:


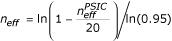


Here, *n*_*eff *_corresponds to the number of randomly aligned sequences with the average number of residue types per position equal to *n*_*eff*_^*PSIC *^(for more details, see [16]).

### Profiles corresponding to fragments of protein structures

We applied our method to compare structure-based predictions of residue probabilities to the actual residue frequencies observed among sequence homologs. For such a comparison, we produced sequence profiles that correspond to fragments of known 3D structures. Briefly, we used a non-redundant set of structures from PDB (minimum 40 residues long, X-ray structure resolution no more than 2.5, NMR structures excluded, no pairs with sequence identity above 20%). SCOP [45,46]. entries classified as membrane proteins or small proteins enriched with disulfide bonds or metal ions were excluded. The final dataset contained 1695 SCOP domains. Starting from the sequence of each domain, PSI-BLAST searches were performed to 5 iterations over the non-redundant NCBI database, with a conservative E-value cutoff of 10^-5^. In the resulting multiple alignments of detected homologs, we purged sequences whose identity to the query was less than 25%, so that only confident sequence homologs were used for profile construction. We split query sequence into fragments of fixed length *F*. For each fragment, we extracted the corresponding segment of the multiple alignment and removed the sequences with deletions (gaps) in this fragment. For a query of length *L *we produced *L*-*F*+1 sub-alignments and derived effective residue counts as described above. In this work, we used the library of profile fragments of length *F *= 6, which provided accurate results when applied to the prediction of local structural environment from a sequence profile [47].

### Prediction of expected residue frequencies from local structural environment

The equilibrium frequency of an amino acid at a position in protein structure reflects the energetic fitness of the sidechain in the local structural environment [38,39]. To estimate these frequencies, we employed the scheme similar to those used to derive statistical or knowledge-based potentials [38-41] or environment-specific substitution tables [42,43]. In brief, we divided structural positions into discrete classes based on local structural environment, and analyzed residue contents for each class of positions in known protein structures. As the characteristics of local structural environment, we used the backbone conformations (*φ *and *ψ *dihedral angles) at the given position and the preceding position, and solvent accessibility of the sidechain at the given position. For a given position we used the partition of Ramachandran plot into 15 (*φ*, *ψ*) classes proposed by Shortle [39]., combined with 3 ranges of relative sidechain solvent accessibility as calculated by the NACCESS package [48] For the position preceding the given, we used a less detailed partition of Ramachandran plot into 6 classes. For each of the resulting 15 × 3 × 6 = 270 classes, we analyzed the set of PDB structures described in the previous section and derived the probabilities of residue types to occur in a class. These probabilities were used as frequency predictions at the structural positions that belong to the class. We assessed consistency of these predictions with residue frequencies in multiple alignments of sequence homologs.

### Pairs of profiles corresponding to pairs of similar structures

As the second application, we estimated statistical significance of similarity between pairs of columns in multiple alignments. Namely, we used pairs of structurally similar proteins (according to the FSSP database [29,30]), produced multiple alignments of their sequence homologs detected by PSI-BLAST, and assessed the consistency between structurally equivalent positions of these multiple alignments.

We chose protein pairs with relatively low sequence identities, where detection of similarity between sequences is not straitforward. We focused on two identity ranges: 25 ± 1% (at the upper bound of twilight zone) and a lower range of 15 ± 1%. From each FSSP family, we extracted the parent sequence and all sequences of a significant structural similarity to the parent (Z-score greater than 5.0), with sequence identity to the parent within a given range. We found totally 494 and 1406 sequence pairs with identities 25 ± 1% and 15 ± 1%, respectively. These numbers were reduced by purging symmetric pairs and manual inspection of the remaining domains for the presence of repeats and low-complexity regions. For further analysis, we used 251 sequence pairs with identity 25 ± 1% and 340 pairs with identity 15 ± 1%, each pair representing a unique FSSP family. For each sequence, we ran 5 iterations of PSI-BLAST 2.2.1 against the NCBI nr database (E-value threshold for inclusion in the next iteration 0.005) and obtained multiple alignments of detected homologs. We then applied a procedure of the alignment processing similar to that implemented in PSI-BLAST [1] In particular, only one copy was retained of any rows that were >97% identical to one another, and the columns with gaps in the first (query) sequence were purged. The resulting multiple alignments were used to calculate P-values for confident structure-based position matches (positions represented as capital letters in FSSP alignments).

### Calculation of solvent accessibility

Solvent accessible surface area (ASA) for the residues of interest was determined using NACCESS package [48], which was applied to PDB structures, with heteroatoms excluded. To determine ASA for carboxyl groups of aspartate and glutamate, the sum of ASA for atoms of these groups was calculated. Residue contacts were determined using default settings of NACCESS.

## Authors' contributions

RS carried out the theoretical considerations, computational experiments, analysis of the results and drafted the manuscript. NG conceived of the study, and participated in its design and coordination. Both authors read and approved the final manuscript.

## Supplementary Material

Additional File 1 "P-value for multivariate Gaussian distribution". Click here for file

Additional File 2 "Upper estimate of P-value for similarity between two alignment columns". Click here for file
