# Supplementary material for: Estimates of statistical significance for comparison of individual positions in multiple sequence alignments
Source: BMC Bioinformatics. 2004 Aug 5;5:106. doi: 10.1186/1471-2105-5-106 (PMC516024; doi:10.1186/1471-2105-5-106)
Supplement: Additional File 1 — "P-value for multivariate Gaussian distribution". [file 1471-2105-5-106-S1.doc]

## Appendix 1. P-value for multivariate Gaussian distribution.

P.d.f. for multivariate Gaussian distribution has the following form:

$\rho\left(x|f\right)=\left(2\pi\right)^{-\frac{d}{2}}\left|\Sigma^{-\frac{1}{2}}\right|\exp\left[-\frac{1}{2}\left(x-\bar{x}\right)^{'}\Sigma^{-1}\left(x-\bar{x}\right)\right]$ (A1)

where $x=\left\{x_{i}\right\}$ is a random *d*-dimensional vector of residue counts of size $\sum_{i} x_{i} = N$, $f$ is emission vector of residue frequencies, $\bar{x} = Nf$ is the mean vector of residue counts, $\Sigma= \left\|cov\left(x_{i},x_{j}\right)\right\|$ is the covariance matrix. Due to fixed size *N*, vector $x$ has (*d*-1) independent components. P-value for the generation of a given residue count vector $x*$ by the emission vector $f$ is, by definition,

$P\left(x*|f\right)=\int_{\rho\left(x|f\right)<\rho\left(x*|f\right)} \rho\left(x|f\right)dx$ (A2)

where the integral is taken over all vectors $x$ with probability density $\rho\left(x|f\right)$ lower than that for the generation of vector $x*$. To calculate this integral, we will apply translation and rotation of coordinate axes ($z=A\left(x-\bar{x}\right)$) and diagonalize quadratic form in the expression for $\rho\left(x|f\right)$:

$\rho\left(x|f\right)=C_{1}\exp\left(-\frac{1}{2}\sum_{i} \lambda_{i}z_{i}^{2}\right)$ (A3)

where $C_{1}=\left(2\pi\right)^{-\frac{d}{2}}\left|\Sigma^{-\frac{1}{2}}\right|\det\left(\frac{\partial x}{\partial z}\right)$ is a constant, $\det\left(\frac{\partial x}{\partial z}\right)$ is Jacobian for the transformation of coordinates, $\lambda_{i}$ are eigenvalues. (In this and further expressions, the exact form of normalizing coefficients *C* is not important for our consideration.) Substitution $t_{i}=\sqrt{\lambda_{i}}z_{i}$ transfroms the integral into

$P\left(x*|f\right)=C_{2}\int_{\sum_{i} t_{i}^{2}>\sum_{i} (t_{i}*)^{2}} \exp\left(-\sum_{i} t_{i}^{2}\right)dt$ (A4)

Here, the integral is taken over the volume outside the sphere in (*d-*1)-dimensional space of independent components; *C*_2_ is a constant. In spheric coordinates, with , this integral has a simple form:

$P\left(x*|f\right)=C_{2}\int_{r^{2}>(r*)^{2}} e^{-r^{2}}r^{d-2}drd\Phi$ (A5)

After integration over the full angle and substitution $\xi=r^{2}$ we have

$P\left(x*|f\right)=C_{2}\frac{\pi^{\frac{d-1}{2}}}{\Gamma\left(\frac{d-1}{2}\right)}\int_{\xi*=(r*)^{2}}^{\infty} e^{-\xi}\xi^{\frac{d-1}{2}-1}d\xi=C_{2}\pi^{\frac{d-1}{2}}\frac{\Gamma\left(\frac{d-1}{2},(r*)^{2}\right)}{\Gamma\left(\frac{d-1}{2}\right)}$ (A6)

where $\Gamma\left(x\right)$ and $\Gamma\left(a,x\right)$are gamma function and incomplete gamma function, respectively. Using the definition of regularized gamma function $Q\left(a,x\right)=\frac{\Gamma\left(a,x\right)}{\Gamma\left(a\right)}$,

$P\left(x*|f\right)=C_{3}Q\left(\frac{d-1}{2},(r*)^{2}\right)$ (A7)

where $(r*)^{2}=\frac{1}{2}\left(x*-Nf\right)^{'}\Sigma^{-1}\left(x*-Nf\right)$. In the case of multivariate Gaussian distribution corresponding to multinomial distribution, covariance matrix $\Sigma= \left\|cov\left(x_{i},x_{j}\right)\right\|$ has a simple form: $cov\left(x_{i},x_{j}\right)=Nf_{i}\left(1-f_{i}\right)$ for *i* = *j* , and $cov\left(x_{i},x_{j}\right)=-Nf_{i}f_{j}$ for *i* ≠ *j*. Expression for $(r*)^{2}$ then transforms into $(r*)^{2}=\frac{1}{2}\sum_{i} \frac{(x_{i}-Nf_{i})^{2}}{Nf_{i}}$.

At the infinity, P-value equals 1, and $Q\left(\frac{d-1}{2},\infty\right)=1$; therefore, in (A7) $C_{3}$ = 1, and

$P\left(x*|f\right)=Q\left(\frac{d-1}{2},\frac{1}{2}\sum_{i} \frac{(x_{i}-Nf_{i})^{2}}{Nf_{i}}\right)$ (A8)
