# Supplementary material for: Estimates of statistical significance for comparison of individual positions in multiple sequence alignments
Source: BMC Bioinformatics. 2004 Aug 5;5:106. doi: 10.1186/1471-2105-5-106 (PMC516024; doi:10.1186/1471-2105-5-106)
Supplement: Additional File 2 — "Upper estimate of P-value for similarity between two alignment columns". [file 1471-2105-5-106-S2.doc]

## Appendix 2. Upper estimate of P-value for similarity between two alignment columns.

For two alignment columns ***m**** and ***n****, we will estimate P-value for the independent generation of these columns by a single emission vector ***f***, with prior distribution of emission vectors $\theta\left(f\right)$

$P\left(m*,n*|\theta\left(f\right)\right)=\int_{\rho\left(m,n|f\right)<\rho\left(m*,n*|f\right)} \rho\left(m,n|f\right)\theta\left(f\right)dm dn df$ (B1)

Here, the integral is calculated over all emission vectors $f$ and over those random residue counts ***m***, ***n***, which have probability density $\rho\left(m,n|f\right)$ lower than that for the generation of vectors ***m****, ***n**** . For each given vector ***f***, the integral over ***m, n*** can be calculated precisely. As follows from Appendix A, P-value for separate generation of each column by an emission vector ***f*** with dimensionality *d* obeys $\chi^{2}$ distribution with (*d*-1) degrees of freedom (formula (A8)). Threfore, the combined distribution for two independently generated random columns is also a $\chi^{2}$, with the number of degrees of freedom (*d*-1) + (*d*-1) = 2(*d*-1). Using notation of Appendix A,

$P\left(m*,n*|f\right)=\int_{\rho\left(m,n|f\right)<\rho\left(m*,n*|f\right)} \rho\left(m|f\right)\rho\left(n|f\right)dm dn =Q\left(d-1,\sum_{i} R_{i}^{2}\right)$ (B2)

where

$R_{i}^{2}=\frac{1}{2}\left(\frac{(m_{i}*-Mf_{i})^{2}}{Mf_{i}}+\frac{(n_{i}*-Nf_{i})^{2}}{Nf_{i}}\right)$ (B3)

We will use prior distribution θ(***f***) that maximizes likelihood for observed alignment columns to be generated by a single emission vector ***f***. Considering θ(***f***) in a simple Gaussian form

$\theta\left(f\right)=\prod_{i} \left(2\pi\hat{\sigma}_{i}^{2}\right)^{-\frac{1}{2}}\exp\left[-\sum_{i} \frac{\left(f_{i}-\hat{f}_{i}\right)^{2}}{2\hat{\sigma}_{i}^{2}}\right]$ (B4)

it is easy to show [49] that

$\hat{f}_{i}=\frac{m_{i}+n_{i}}{M+N}$, $\hat{\sigma}_{i}^{2}=\frac{1}{2}\left[\frac{\left(n_{i}-N\hat{f}_{i}\right)^{2}}{N}+\frac{\left(m_{i}-M\hat{f}_{i}\right)^{2}}{M}\right]$ (B5)

Formula (B2) and expression (B4) for the prior transform (B1) into

$P=\prod_{i} \left(2\pi\hat{\sigma}_{i}^{2}\right)^{-\frac{1}{2}}\int_{\sum_{i} f_{i}=1} Q\left(d-1,\sum_{i} R_{i}^{2}\right) \exp\left[-\sum_{i} \frac{\left(f_{i}-\hat{f}_{i}\right)^{2}}{2\hat{\sigma}_{i}^{2}}\right]df$ (B6)

Analytic calculation of this integral is problematic, and we will estimate its approximate value using two observations. First, the argument of the regularized gamma function is the sum of partial functions of individual emission frequencies *f_i_*, which reaches its minimum at ***f*^(0)^** = {*f_i_*^(0)^}:

$f_{i}^{(0)}=\alpha\left(\sqrt{1/\alpha+1}-1\right)$, $\alpha=\frac{\frac{n_{i}^{2}}{N}+\frac{m_{i}^{2}}{M}}{M+N-2(n_{i}+m_{i})}$ (B7)

Second, function $Q\left(a,x\right)=\frac{\Gamma\left(a,x\right)}{\Gamma\left(a\right)}$ monotonically decreases with *x* from $Q\left(a,0\right)=1$ to $Q\left(a,\infty\right)=0$. Therefore, the regularized gamma function *Q* in (B6) reaches its single maximum *Q*_max_ at ***f*^(0)^** = {*f_i_*^(0)^} and rapidly decreases to zero outside the vicinity of ***f*^(0)^**. Based on this result, we determine volume Ω around ***f*^(0)^** where $Q\left(d-1,\sum_{i} R_{i}^{2}\right)$ is still comparable with *Q*_max_, and calculate an approximate upper estimate of integral (B6) as

$P=Q_{\max}\prod_{i} \left(2\pi\hat{\sigma}_{i}^{2}\right)^{-\frac{1}{2}}\int_{\Omega} \exp\left[-\sum_{i} \frac{\left(f_{i}-\hat{f}_{i}\right)^{2}}{2\hat{\sigma}_{i}^{2}}\right]df$ (B8)

We define volume Ω as a parallelepiped and estimate the location of its edges. First, we consider $Q\left(d-1,\sum_{i} R_{i}^{2}\right)$ in (B6) and estimate the characteristic distance Δ from the maximum point *x*^(0)^=$\sum_{i} R_{i}^{2}\left(f_{i}^{(0)}\right)$ for which $Q\left(d-1,x^{(0)}+\Delta\right)$<<$Q\left(d-1,x^{(0)}\right)$. Specifically, we approximate $Q\left(d-1,x\right)$ with tangent at $x^{(0)}$ and find the point of intersection between the tangent and abscissa:

$Q\left(d-1,x^{(0)}\right)-\frac{\partial Q\left(d-1,x=x_{0}\right)}{\partial x}\Delta=0$ (B9)

Given that $\frac{\partial Q\left(a,x\right)}{\partial x}=-\frac{x^{a-1}e^{-x}}{\Gamma(a)}$, we can estimate distance Δ as

$\Delta=\frac{\Gamma\left(d-1,x^{(0)}\right)e^{-x^{(0)}}}{\left(x^{(0)}\right)^{d-2}}$ (B10)

Based on this estimate, we determine borders of volume Ω in the space of emission vectors. For simplicity, we defined Ω as parallelepiped {*f_i_*^(1)^ < *f_i_* < *f_i_*^(2)^}, *i=1,d*-1, where limits *f_i_*^(1,2)^ are determined from equation

$\sum_{i} R_{i}^{2}(f_{i})=\sum_{i} R_{i}^{2}\left(f_{i}^{(0)}\right)+\Delta$ (B11)

Using expressions for $R_{i}^{2}(f_{i})$ from (B3), we get the borders of Ω as

$f_{i}^{(1,2)}=\frac{b\pm\sqrt{b^{2}-ac}}{a}$ (B12)

where $a=M+N+\sum_{i} R_{i}^{2}\left(f_{i}^{(0)}\right)+2\Delta$, $b=m_{1}*+n_{1}*+\frac{1}{2}\sum_{i} R_{i}^{2}\left(f_{i}^{(0)}\right)+\Delta$, $c=\frac{\left(m_{i}*\right)^{2}}{M}+\frac{\left(n_{i}*\right)^{2}}{N}$.

Having Ω and using the definition of error function *erf*(*x*), we calculate an approximate upper estimate for P-value (B8) as

$P=Q\left(d-1,\frac{1}{2}\sum_{i} R_{i}^{2}\left(f_{i}^{(0)}\right)\right)\prod_{i} \left[\frac{1}{2}\left(erf\left(\frac{f_{i}^{(2)}-\hat{f}_{i}}{\sqrt{2\hat{\sigma}_{i}^{2}}}\right)-erf\left(\frac{f_{i}^{(1)}-\hat{f}_{i}}{\sqrt{2\hat{\sigma}_{i}^{2}}}\right)\right)\right]$ (B13)

where $R_{i}^{2}\left(f_{i}^{(0)}\right)$is defined by (B3) and (B7), $\hat{f}_{i}$ and $\hat{\sigma}_{i}^{2}$ are defined by (B5), and $f_{i}^{(1,2)}$ are defined by (B12).
